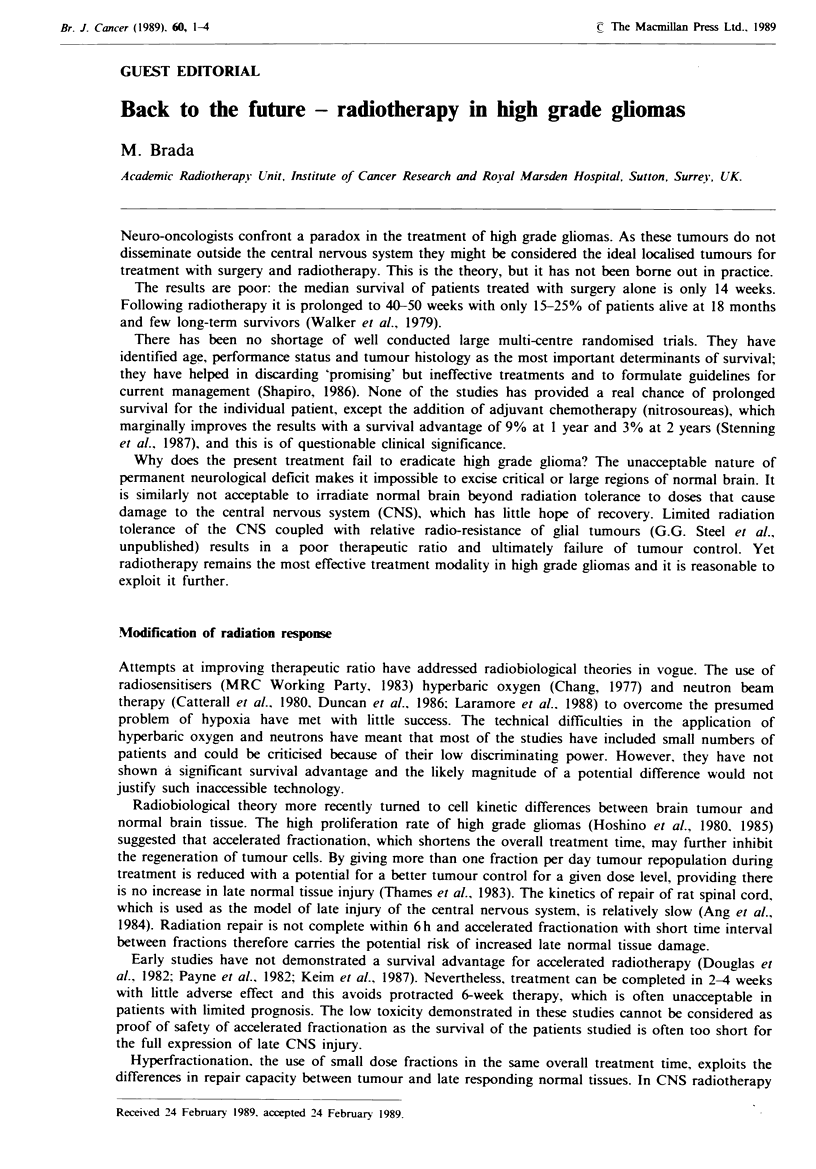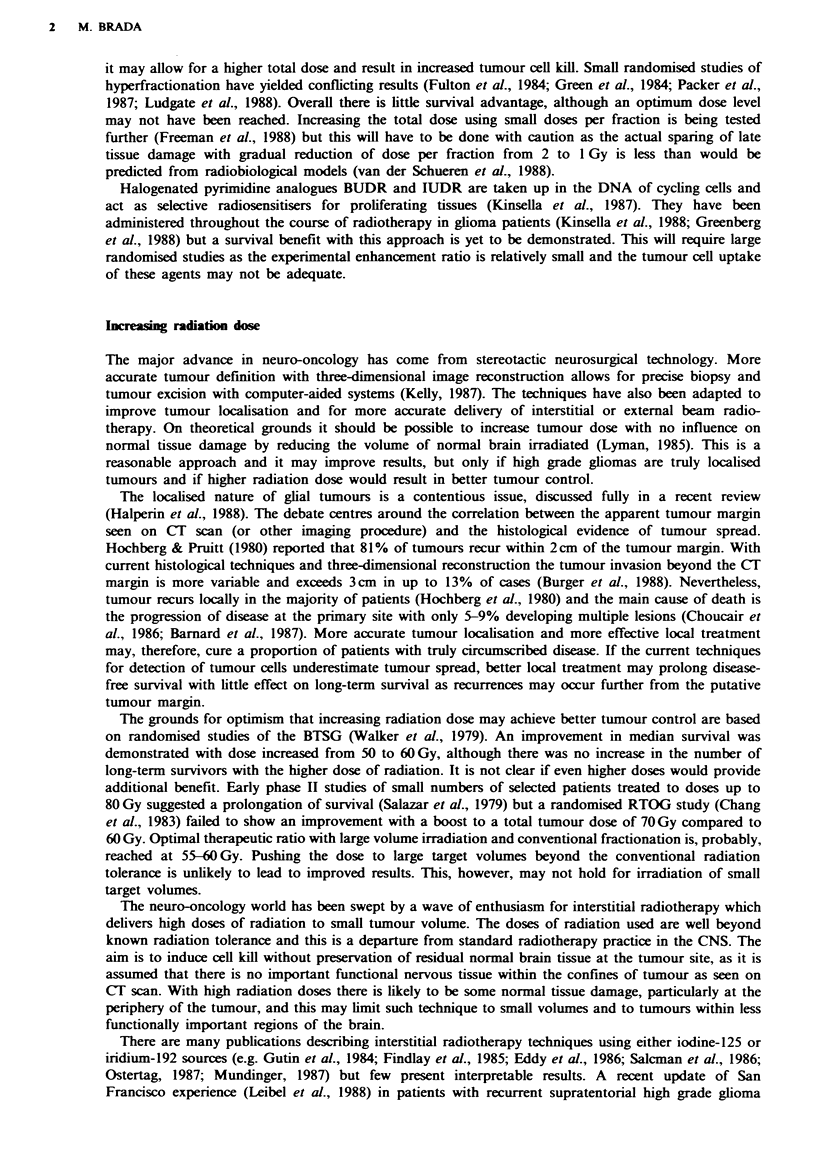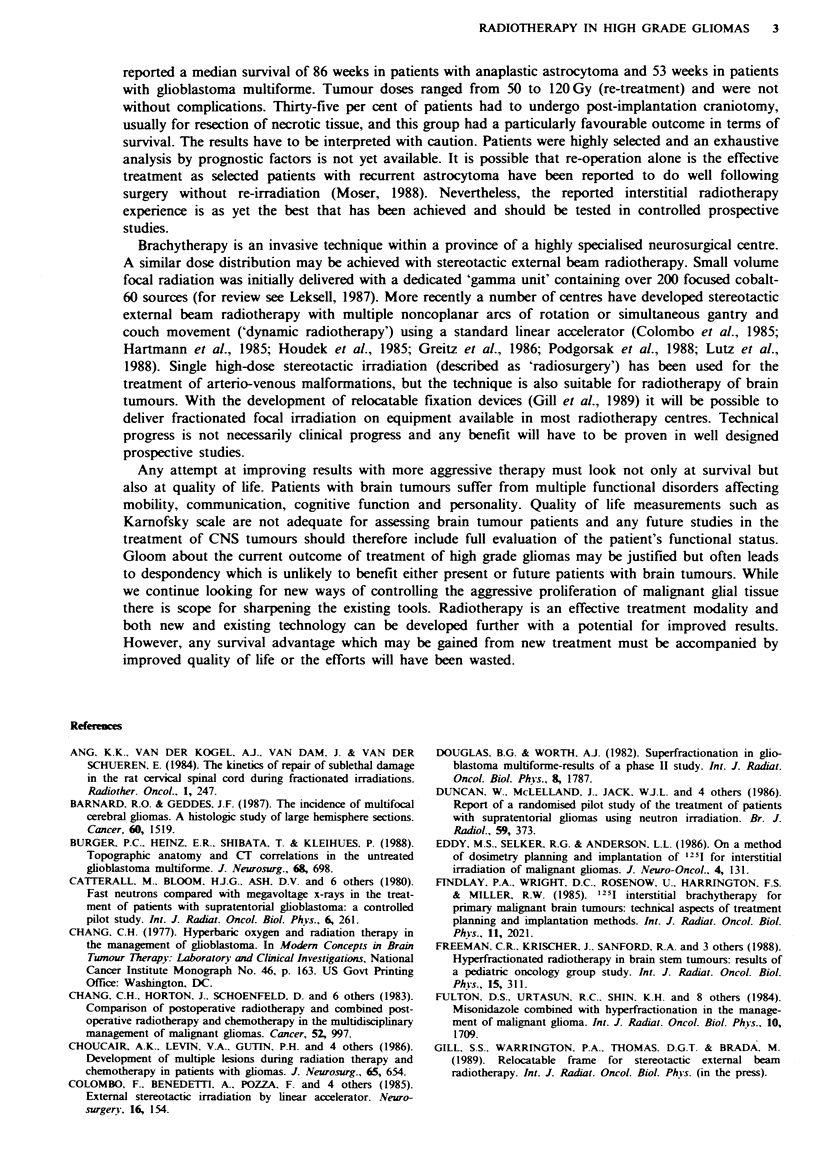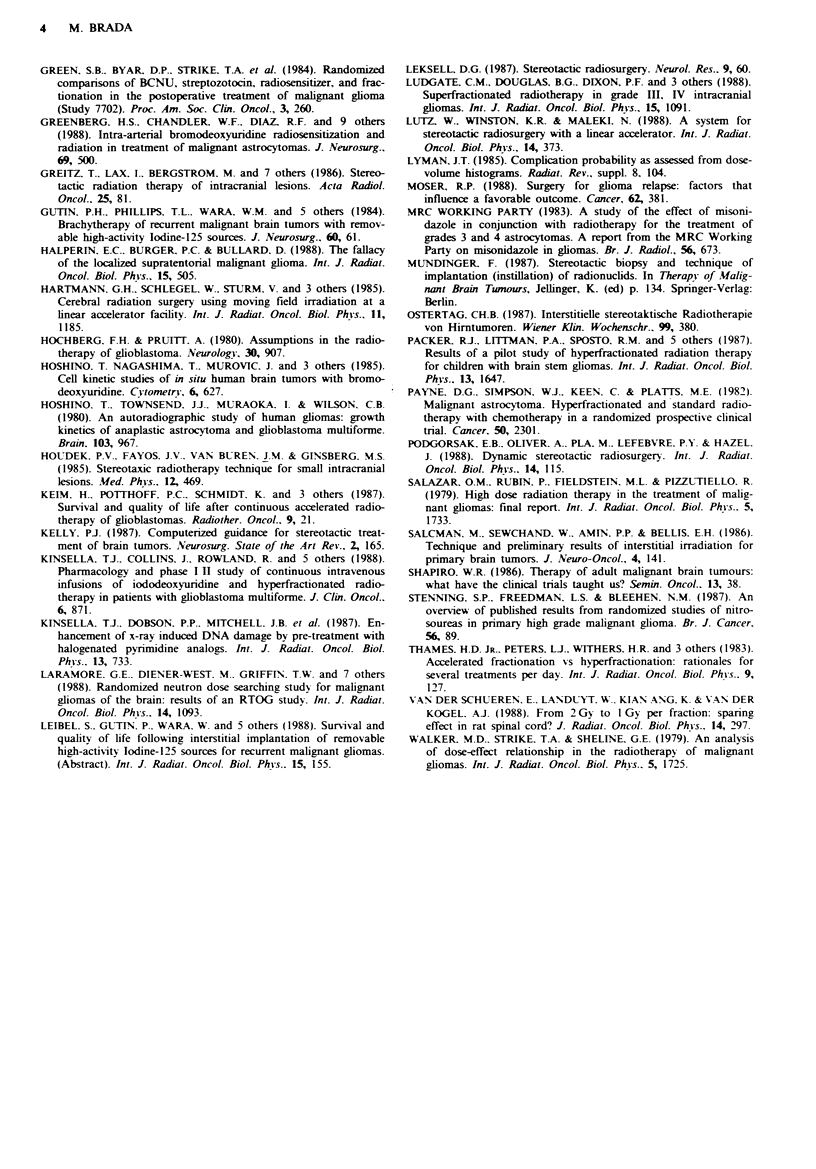# Back to the future--radiotherapy in high grade gliomas.

**DOI:** 10.1038/bjc.1989.207

**Published:** 1989-07

**Authors:** M. Brada

**Affiliations:** Academic Radiotherapy Unit, Institute of Cancer Research, Sutton, Surrey, UK.


Br. J. Cancer (1989). 60, 1–4 © The Macmillan Press Ltd., 1989

GUEST EDITORIAL

### Back to the future – radiotherapy in high grade gliomas

M. Brada

*Academic Radiotherapy Unit, Institute of Cancer Research and Royal Marsden Hospital, Sutton, Surrey, UK.*

Neuro-oncologists confront a paradox in the treatment of high grade gliomas. As these tumours do not disseminate outside the central nervous system they might be considered the ideal localised tumours for treatment with surgery and radiotherapy. This is the theory, but it has not been borne out in practice.

The results are poor: the median survival of patients treated with surgery alone is only 14 weeks. Following radiotherapy it is prolonged to 40–50 weeks with only 15–25% of patients alive at 18 months and few long-term survivors (Walker *et al.*, 1979).

There has been no shortage of well conducted large multi-centre randomised trials. They have identified age, performance status and tumour histology as the most important determinants of survival; they have helped in discarding 'promising' but ineffective treatments and to formulate guidelines for current management (Shapiro, 1986). None of the studies has provided a real chance of prolonged survival for the individual patient, except the addition of adjuvant chemotherapy (nitrosoureas), which marginally improves the results with a survival advantage of 9% at 1 year and 3% at 2 years (Stenning *et al.*, 1987), and this is of questionable clinical significance.

Why does the present treatment fail to eradicate high grade glioma? The unacceptable nature of permanent neurological deficit makes it impossible to excise critical or large regions of normal brain. It is similarly not acceptable to irradiate normal brain beyond radiation tolerance to doses that cause damage to the central nervous system (CNS), which has little hope of recovery. Limited radiation tolerance of the CNS coupled with relative radio-resistance of glial tumours (G.G. Steel *et al.*, unpublished) results in a poor therapeutic ratio and ultimately failure of tumour control. Yet radiotherapy remains the most effective treatment modality in high grade gliomas and it is reasonable to exploit it further.

### Modification of radiation response

Attempts at improving therapeutic ratio have addressed radiobiological theories in vogue. The use of radiosensitisers (MRC Working Party, 1983) hyperbaric oxygen (Chang, 1977) and neutron beam therapy (Catterall *et al.*, 1980, Duncan *et al.*, 1986; Laramore *et al.*, 1988) to overcome the presumed problem of hypoxia have met with little success. The technical difficulties in the application of hyperbaric oxygen and neutrons have meant that most of the studies have included small numbers of patients and could be criticised because of their low discriminating power. However, they have not shown a significant survival advantage and the likely magnitude of a potential difference would not justify such inaccessible technology.

Radiobiological theory more recently turned to cell kinetic differences between brain tumour and normal brain tissue. The high proliferation rate of high grade gliomas (Hoshino *et al.*, 1980, 1985) suggested that accelerated fractionation, which shortens the overall treatment time, may further inhibit the regeneration of tumour cells. By giving more than one fraction per day tumour repopulation during treatment is reduced with a potential for a better tumour control for a given dose level, providing there is no increase in late normal tissue injury (Thames *et al.*, 1983). The kinetics of repair of rat spinal cord, which is used as the model of late injury of the central nervous system, is relatively slow (Ang *et al.*, 1984). Radiation repair is not complete within 6 h and accelerated fractionation with short time interval between fractions therefore carries the potential risk of increased late normal tissue damage.

Early studies have not demonstrated a survival advantage for accelerated radiotherapy (Douglas *et al.*, 1982; Payne *et al.*, 1982; Keim *et al.*, 1987). Nevertheless, treatment can be completed in 2–4 weeks with little adverse effect and this avoids protracted 6-week therapy, which is often unacceptable in patients with limited prognosis. The low toxicity demonstrated in these studies cannot be considered as proof of safety of accelerated fractionation as the survival of the patients studied is often too short for the full expression of late CNS injury.

Hyperfractionation, the use of small dose fractions in the same overall treatment time, exploits the differences in repair capacity between tumour and late responding normal tissues. In CNS radiotherapy

Received 24 February 1989, accepted 24 February 1989.

it may allow for a higher total dose and result in increased tumour cell kill. Small randomised studies of hyperfractionation have yielded conflicting results (Fulton *et al.*, 1984; Green *et al.*, 1984; Packer *et al.*, 1987; Ludgate *et al.*, 1988). Overall there is little survival advantage, although an optimum dose level may not have been reached. Increasing the total dose using small doses per fraction is being tested further (Freeman *et al.*, 1988) but this will have to be done with caution as the actual sparing of late tissue damage with gradual reduction of dose per fraction from 2 to 1 Gy is less than would be predicted from radiobiological models (van der Schueren *et al.*, 1988).

Halogenated pyrimidine analogues BUDR and IUDR are taken up in the DNA of cycling cells and act as selective radiosensitisers for proliferating tissues (Kinsella *et al.*, 1987). They have been administered throughout the course of radiotherapy in glioma patients (Kinsella *et al.*, 1988; Greenberg *et al.*, 1988) but a survival benefit with this approach is yet to be demonstrated. This will require large randomised studies as the experimental enhancement ratio is relatively small and the tumour cell uptake of these agents may not be adequate.

**Increasing radiation dose**

The major advance in neuro-oncology has come from stereotactic neurosurgical technology. More accurate tumour definition with three-dimensional image reconstruction allows for precise biopsy and tumour excision with computer-aided systems (Kelly, 1987). The techniques have also been adapted to improve tumour localisation and for more accurate delivery of interstitial or external beam radiotherapy. On theoretical grounds it should be possible to increase tumour dose with no influence on normal tissue damage by reducing the volume of normal brain irradiated (Lyman, 1985). This is a reasonable approach and it may improve results, but only if high grade gliomas are truly localised tumours and if higher radiation dose would result in better tumour control.

The localised nature of glial tumours is a contentious issue, discussed fully in a recent review (Halperin *et al.*, 1988). The debate centres around the correlation between the apparent tumour margin seen on CT scan (or other imaging procedure) and the histological evidence of tumour spread. Hochberg & Pruitt (1980) reported that 81% of tumours recur within 2 cm of the tumour margin. With current histological techniques and three-dimensional reconstruction the tumour invasion beyond the CT margin is more variable and exceeds 3 cm in up to 13% of cases (Burger *et al.*, 1988). Nevertheless, tumour recurs locally in the majority of patients (Hochberg *et al.*, 1980) and the main cause of death is the progression of disease at the primary site with only 5–9% developing multiple lesions (Choucair *et al.*, 1986; Barnard *et al.*, 1987). More accurate tumour localisation and more effective local treatment may, therefore, cure a proportion of patients with truly circumscribed disease. If the current techniques for detection of tumour cells underestimate tumour spread, better local treatment may prolong disease-free survival with little effect on long-term survival as recurrences may occur further from the putative tumour margin.

The grounds for optimism that increasing radiation dose may achieve better tumour control are based on randomised studies of the BTSG (Walker *et al.*, 1979). An improvement in median survival was demonstrated with dose increased from 50 to 60 Gy, although there was no increase in the number of long-term survivors with the higher dose of radiation. It is not clear if even higher doses would provide additional benefit. Early phase II studies of small numbers of selected patients treated to doses up to 80 Gy suggested a prolongation of survival (Salazar *et al.*, 1979) but a randomised RTOG study (Chang *et al.*, 1983) failed to show an improvement with a boost to a total tumour dose of 70 Gy compared to 60 Gy. Optimal therapeutic ratio with large volume irradiation and conventional fractionation is, probably, reached at 55–60 Gy. Pushing the dose to large target volumes beyond the conventional radiation tolerance is unlikely to lead to improved results. This, however, may not hold for irradiation of small target volumes.

The neuro-oncology world has been swept by a wave of enthusiasm for interstitial radiotherapy which delivers high doses of radiation to small tumour volume. The doses of radiation used are well beyond known radiation tolerance and this is a departure from standard radiotherapy practice in the CNS. The aim is to induce cell kill without preservation of residual normal brain tissue at the tumour site, as it is assumed that there is no important functional nervous tissue within the confines of tumour as seen on CT scan. With high radiation doses there is likely to be some normal tissue damage, particularly at the periphery of the tumour, and this may limit such technique to small volumes and to tumours within less functionally important regions of the brain.

There are many publications describing interstitial radiotherapy techniques using either iodine-125 or iridium-192 sources (e.g. Gutin *et al.*, 1984; Findlay *et al.*, 1985; Eddy *et al.*, 1986; Salcman *et al.*, 1986; Ostertag, 1987; Mundinger, 1987) but few present interpretable results. A recent update of San Francisco experience (Leibel *et al.*, 1988) in patients with recurrent supratentorial high grade glioma reported a median survival of 86 weeks in patients with anaplastic astrocytoma and 53 weeks in patients with glioblastoma multiforme. Tumour doses ranged from 50 to 120 Gy (re-treatment) and were not without complications. Thirty-five per cent of patients had to undergo post-implantation craniotomy, usually for resection of necrotic tissue, and this group had a particularly favourable outcome in terms of survival. The results have to be interpreted with caution. Patients were highly selected and an exhaustive analysis by prognostic factors is not yet available. It is possible that re-operation alone is the effective treatment as selected patients with recurrent astrocytoma have been reported to do well following surgery without re-irradiation (Moser, 1988). Nevertheless, the reported interstitial radiotherapy experience is as yet the best that has been achieved and should be tested in controlled prospective studies.

Brachytherapy is an invasive technique within a province of a highly specialised neurosurgical centre. A similar dose distribution may be achieved with stereotactic external beam radiotherapy. Small volume focal radiation was initially delivered with a dedicated 'gamma unit' containing over 200 focused cobalt-60 sources (for review see Leksell, 1987). More recently a number of centres have developed stereotactic external beam radiotherapy with multiple noncoplanar arcs of rotation or simultaneous gantry and couch movement ('dynamic radiotherapy') using a standard linear accelerator (Colombo *et al.*, 1985; Hartmann *et al.*, 1985; Houdek *et al.*, 1985; Greitz *et al.*, 1986; Podgorsak *et al.*, 1988; Lutz *et al.*, 1988). Single high-dose stereotactic irradiation (described as 'radiosurgery') has been used for the treatment of arterio-venous malformations, but the technique is also suitable for radiotherapy of brain tumours. With the development of relocatable fixation devices (Gill *et al.*, 1989) it will be possible to deliver fractionated focal irradiation on equipment available in most radiotherapy centres. Technical progress is not necessarily clinical progress and any benefit will have to be proven in well designed prospective studies.

Any attempt at improving results with more aggressive therapy must look not only at survival but also at quality of life. Patients with brain tumours suffer from multiple functional disorders affecting mobility, communication, cognitive function and personality. Quality of life measurements such as Karnofsky scale are not adequate for assessing brain tumour patients and any future studies in the treatment of CNS tumours should therefore include full evaluation of the patient's functional status. Gloom about the current outcome of treatment of high grade gliomas may be justified but often leads to despondency which is unlikely to benefit either present or future patients with brain tumours. While we continue looking for new ways of controlling the aggressive proliferation of malignant glial tissue there is scope for sharpening the existing tools. Radiotherapy is an effective treatment modality and both new and existing technology can be developed further with a potential for improved results. However, any survival advantage which may be gained from new treatment must be accompanied by improved quality of life or the efforts will have been wasted.

**References**

ANG, K.K., VAN DER KOGEL, A.J., VAN DAM, J. & VAN DER SCHUEREN, E. (1984). The kinetics of repair of sublethal damage in the rat cervical spinal cord during fractionated irradiations. *Radiother. Oncol.*, **1**, 247.

BARNARD, R.O. & GEDDES, J.F. (1987). The incidence of multifocal cerebral gliomas. A histologic study of large hemisphere sections. *Cancer*, **60**, 1519.

BURGER, P.C., HEINZ, E.R., SHIBATA, T. & KLEIHUES, P. (1988). Topographic anatomy and CT correlations in the untreated glioblastoma multiforme. *J. Neurosurg.*, **68**, 698.

CATTERALL, M., BLOOM, H.J.G., ASH, D.V. and 6 others (1980). Fast neutrons compared with megavoltage x-rays in the treatment of patients with supratentorial glioblastoma: a controlled pilot study. *Int. J. Radiat. Oncol. Biol. Phys.*, **6**, 261.

CHANG, C.H. (1977). Hyperbaric oxygen and radiation therapy in the management of glioblastoma. In *Modern Concepts in Brain Tumour Therapy: Laboratory and Clinical Investigations*, National Cancer Institute Monograph No. 46, p. 163. US Govt Printing Office: Washington, DC.

CHANG, C.H., HORTON, J., SCHOENFELD, D. and 6 others (1983). Comparison of postoperative radiotherapy and combined post-operative radiotherapy and chemotherapy in the multidisciplinary management of malignant gliomas. *Cancer*, **52**, 997.

CHOUCAIR, A.K., LEVIN, V.A., GUTIN, P.H. and 4 others (1986). Development of multiple lesions during radiation therapy and chemotherapy in patients with gliomas. *J. Neurosurg.*, **65**, 654.

COLOMBO, F., BENEDETTI, A., POZZA, F. and 4 others (1985). External stereotactic irradiation by linear accelerator. *Neurosurgery*, **16**, 154.

DOUGLAS, B.G. & WORTH, A.J. (1982). Superfractionation in glioblastoma multiforme-results of a phase II study. *Int. J. Radiat. Oncol. Biol. Phys.*, **8**, 1787.

DUNCAN, W., McLELLAND, J., JACK, W.J.L. and 4 others (1986). Report of a randomised pilot study of the treatment of patients with supratentorial gliomas using neutron irradiation. *Br. J. Radiol.*, **59**, 373.

EDDY, M.S., SELKER, R.G. & ANDERSON, L.L. (1986). On a method of dosimetry planning and implantation of $^{125}$I for interstitial irradiation of malignant gliomas. *J. Neuro-Oncol.*, **4**, 131.

FINDLAY, P.A., WRIGHT, D.C., ROSENOW, U., HARRINGTON, F.S. & MILLER, R.W. (1985). $^{125}$I interstitial brachytherapy for primary malignant brain tumours: technical aspects of treatment planning and implantation methods. *Int. J. Radiat. Oncol. Biol. Phys.*, **11**, 2021.

FREEMAN, C.R., KRISCHER, J., SANFORD, R.A. and 3 others (1988). Hyperfractionated radiotherapy in brain stem tumours: results of a pediatric oncology group study. *Int. J. Radiat. Oncol. Biol. Phys.*, **15**, 311.

FULTON, D.S., URTASUN, R.C., SHIN, K.H. and 8 others (1984). Misonidazole combined with hyperfractionation in the management of malignant glioma. *Int. J. Radiat. Oncol. Biol. Phys.*, **10**, 1709.

GILL, S.S., WARRINGTON, P.A., THOMAS, D.G.T. & BRADA, M. (1989). Relocatable frame for stereotactic external beam radiotherapy. *Int. J. Radiat. Oncol. Biol. Phys.* (in the press).

GREEN, S.B., BYAR, D.P., STRIKE, T.A. *et al.* (1984). Randomized comparisons of BCNU, streptozotocin, radiosensitizer, and fractionation in the postoperative treatment of malignant glioma (Study 7702). *Proc. Am. Soc. Clin. Oncol.*, **3**, 260.

GREENBERG, H.S., CHANDLER, W.F., DIAZ, R.F. and 9 others (1988). Intra-arterial bromodeoxyuridine radiosensitization and radiation in treatment of malignant astrocytomas. *J. Neurosurg.*, **69**, 500.

GREITZ, T., LAX, I., BERGSTROM, M. and 7 others (1986). Stereotactic radiation therapy of intracranial lesions. *Acta Radiol. Oncol.*, **25**, 81.

GUTIN, P.H., PHILLIPS, T.L., WARA, W.M. and 5 others (1984). Brachytherapy of recurrent malignant brain tumors with removable high-activity Iodine-125 sources. *J. Neurosurg.*, **60**, 61.

HALPERIN, E.C., BURGER, P.C. & BULLARD, D. (1988). The fallacy of the localized supratentorial malignant glioma. *Int. J. Radiat. Oncol. Biol. Phys.*, **15**, 505.

HARTMANN, G.H., SCHLEGEL, W., STURM, V. and 3 others (1985). Cerebral radiation surgery using moving field irradiation at a linear accelerator facility. *Int. J. Radiat. Oncol. Biol. Phys.*, **11**, 1185.

HOCHBERG, F.H. & PRUITT, A. (1980). Assumptions in the radiotherapy of glioblastoma. *Neurology*, **30**, 907.

HOSHINO, T. NAGASHIMA, T., MUROVIC, J. and 3 others (1985). Cell kinetic studies of *in situ* human brain tumors with bromodeoxyuridine. *Cytometry*, **6**, 627.

HOSHINO, T., TOWNSEND, J.J., MURAOKA, I. & WILSON, C.B. (1980). An autoradiographic study of human gliomas: growth kinetics of anaplastic astrocytoma and glioblastoma multiforme. *Brain*, **103**, 967.

HOUDEK, P.V., FAYOS, J.V., VAN BUREN, J.M. & GINSBERG, M.S. (1985). Stereotaxic radiotherapy technique for small intracranial lesions. *Med. Phys.*, **12**, 469.

KEIM, H., POTTHOFF, P.C., SCHMIDT, K. and 3 others (1987). Survival and quality of life after continuous accelerated radiotherapy of glioblastomas. *Radiother. Oncol.*, **9**, 21.

KELLY, P.J. (1987). Computerized guidance for stereotactic treatment of brain tumors. *Neurosurg. State of the Art Rev.*, **2**, 165.

KINSELLA, T.J., COLLINS, J., ROWLAND, R. and 5 others (1988). Pharmacology and phase I II study of continuous intravenous infusions of iododeoxyuridine and hyperfractionated radiotherapy in patients with glioblastoma multiforme. *J. Clin. Oncol.*, **6**, 871.

KINSELLA, T.J., DOBSON, P.P., MITCHELL, J.B. *et al.* (1987). Enhancement of x-ray induced DNA damage by pre-treatment with halogenated pyrimidine analogs. *Int. J. Radiat. Oncol. Biol. Phys.*, **13**, 733.

LARAMORE, G.E., DIENER-WEST, M., GRIFFIN, T.W. and 7 others (1988). Randomized neutron dose searching study for malignant gliomas of the brain: results of an RTOG study. *Int. J. Radiat. Oncol. Biol. Phys.*, **14**, 1093.

LEIBEL, S., GUTIN, P., WARA, W. and 5 others (1988). Survival and quality of life following interstitial implantation of removable high-activity Iodine-125 sources for recurrent malignant gliomas. (Abstract). *Int. J. Radiat. Oncol. Biol. Phys.*, **15**, 155.

LEKSELL, D.G. (1987). Stereotactic radiosurgery. *Neurol. Res.*, **9**, 60.

LUDGATE, C.M., DOUGLAS, B.G., DIXON, P.F. and 3 others (1988). Superfractionated radiotherapy in grade III, IV intracranial gliomas. *Int. J. Radiat. Oncol. Biol. Phys.*, **15**, 1091.

LUTZ, W., WINSTON, K.R. & MALEKI, N. (1988). A system for stereotactic radiosurgery with a linear accelerator. *Int. J. Radiat. Oncol. Biol. Phys.*, **14**, 373.

LYMAN, J.T. (1985). Complication probability as assessed from dose-volume histograms. *Radiat. Rev.*, suppl. 8, 104.

MOSER, R.P. (1988). Surgery for glioma relapse: factors that influence a favorable outcome. *Cancer*, **62**, 381.

MRC WORKING PARTY (1983). A study of the effect of misonidazole in conjunction with radiotherapy for the treatment of grades 3 and 4 astrocytomas. A report from the MRC Working Party on misonidazole in gliomas. *Br. J. Radiol.*, **56**, 673.

MUNDINGER, F. (1987). Stereotactic biopsy and technique of implantation (instillation) of radionuclids. In *Therapy of Malignant Brain Tumours*, Jellinger, K. (ed) p. 134. Springer-Verlag: Berlin.

OSTERTAG, CH.B. (1987). Interstitielle stereotaktische Radiotherapie von Hirntumoren. *Wiener Klin. Wochenschr.*, **99**, 380.

PACKER, R.J., LITTMAN, P.A., SPOSTO, R.M. and 5 others (1987). Results of a pilot study of hyperfractionated radiation therapy for children with brain stem gliomas. *Int. J. Radiat. Oncol. Biol. Phys.*, **13**, 1647.

PAYNE, D.G., SIMPSON, W.J., KEEN, C. & PLATTS, M.E. (1982). Malignant astrocytoma. Hyperfractionated and standard radiotherapy with chemotherapy in a randomized prospective clinical trial. *Cancer*, **50**, 2301.

PODGORSAK, E.B., OLIVER, A., PLA, M., LEFEBVRE, P.Y. & HAZEL, J. (1988). Dynamic stereotactic radiosurgery. *Int. J. Radiat. Oncol. Biol. Phys.*, **14**, 115.

SALAZAR, O.M., RUBIN, P., FIELDSTEIN, M.L. & PIZZUTIELLO, R. (1979). High dose radiation therapy in the treatment of malignant gliomas: final report. *Int. J. Radiat. Oncol. Biol. Phys.*, **5**, 1733.

SALCMAN, M., SEWCHAND, W., AMIN, P.P. & BELLIS, E.H. (1986). Technique and preliminary results of interstitial irradiation for primary brain tumors. *J. Neuro-Oncol.*, **4**, 141.

SHAPIRO, W.R. (1986). Therapy of adult malignant brain tumours: what have the clinical trials taught us? *Semin. Oncol.*, **13**, 38.

STENNING, S.P., FREEDMAN, L.S. & BLEEHEN, N.M. (1987). An overview of published results from randomized studies of nitrosoureas in primary high grade malignant glioma. *Br. J. Cancer*, **56**, 89.

THAMES, H.D. JR., PETERS, L.J., WITHERS, H.R. and 3 others (1983). Accelerated fractionation vs hyperfractionation: rationales for several treatments per day. *Int. J. Radiat. Oncol. Biol. Phys.*, **9**, 127.

VAN DER SCHUEREN, E., LANDUYT, W., KIAN ANG, K. & VAN DER KOGEL, A.J. (1988). From 2 Gy to 1 Gy per fraction: sparing effect in rat spinal cord? *J. Radiat. Oncol. Biol. Phys.*, **14**, 297.

WALKER, M.D., STRIKE, T.A. & SHELINE, G.E. (1979). An analysis of dose-effect relationship in the radiotherapy of malignant gliomas. *Int. J. Radiat. Oncol. Biol. Phys.*, **5**, 1725.